# Metformin inhibits proliferation of human keratinocytes through a mechanism associated with activation of the MAPK signaling pathway

**DOI:** 10.3892/etm.2013.1416

**Published:** 2013-11-19

**Authors:** WEINING LI, WEIYUAN MA, HUA ZHONG, WENBIN LIU, QING SUN

**Affiliations:** 1 Department of Dermatology, Qilu Hospital, Shandong University, Jinan, Shandong 250012, P.R. China; 2 Department of Dermatology, Affiliated Hospital of Shandong University of Traditional Chinese Medicine, Jinan, Shandong 250012, P.R. China

**Keywords:** metformin, HaCaT cells, proliferation, psoriasis, adenosine monophosphate-activated protein kinase, extracellular signal-related kinase 1/2

## Abstract

In the present study, the effects of metformin on the proliferation of human immortalized keratinocytes (HaCaTs) and the underlying mechanisms were investigated. HaCaT cells in the logarithmic growth phase were treated with 50 mM metformin for 24, 48 and 72 h. Cell morphology after 24 h of treatment was observed under a microscope. Cell proliferation was detected using a colorimetric cell proliferation and cytotoxicity assay kit. Western blot analyses were performed to detect the protein phosphorylation levels of adenosine monophosphate-activated protein kinase (AMPK) and extracellular signal-related kinase 1/2 (ERK1/2). Metformin treatment resulted in morphological changes of the HaCaT cells. The survival rates of HaCaT cells treated with metformin were 36.18, 12.70 and 10.12% at 24, 48 and 72 h, respectively. As the treatment time extended, the survival rates of HaCaT cells decreased. Western blot analysis results showed that the mean level of phosphorylated (p)-AMPK in the HaCaT cells without metformin treatment was 2.856±0.323. However, the mean p-AMPK level following metformin treatment for 24 h increased to 5.198±0.625, indicating a significant difference between these two groups (P<0.05). The mean absorbance ratio of p-ERK1/2 was 7.550±1.087 for the untreated cells, but the levels in cells following metformin treatment for 24 h increased to 10.430±1.217, indicating a significant difference between the two groups (P<0.05). In conclusion, metformin treatment upregulated the levels of p-AMPK and p-ERK1/2 in HaCaT cells, and significantly inhibited HaCaT cell proliferation *in vitro* by a mechanism associated with activation of the mitogen-activated protein kinase signaling pathway.

## Introduction

Psoriasis is a chronic inflammatory skin disease with genetic predisposition (1–4), characterized by the hyperproliferation and abnormal differentiation of epidermal keratinocytes (5). Inhibition of the excessive proliferation of keratinocytes is the main treatment method of psoriasis (6). HaCaTs are immortalized cell lines derived from keratinocytes in normal adult skin and the excessive proliferation of these keratinocytes results in psoriatic lesions (7). Therefore, HaCaT cells have been widely used as an *in vitro* model for the study of anti-psoriasis agents (8,9).

Metformin is an insulin sensitizer, it is the first-line treatment method for type II diabetes and was recommended by the American Diabetes Association in 2012 for its hypoglycemic effects and ability to reduce cardiovascular morbidity and mortality. In addition, metformin rarely causes lactic acidosis (10,11). Previous studies have shown that metformin inhibits cell growth and proliferation of a number of types of cancer, including liver, colon and prostate cancer (12–14). However, whether metformin inhibits the proliferation of HaCaT cells has, to the best of our knowledge, not been studied.

The mitogen-activated protein kinase (MAPK) signaling pathway is important for the proliferation of HaCaT cells. It has been reported that metformin activates adenosine monophosphate-activated protein kinase (AMPK) in breast cancer MCF-7 cells, inhibits the mTOR signaling pathway to reduce protein translation initiation and decreases cell proliferation (15). Following metformin treatment in ovarian cancer cells, the AMPK signaling pathway is activated and phosphorylated (p)-AMPK protein expression is increased. This results in the inhibition of proliferation-associated protein molecule synthesis and thus suppression of ovarian cancer cell proliferation (16). Metformin also activates AMPK to reduce proliferative signaling in tumor cells and provide direct anti-tumor effects (17).

In the present study, the effects of metformin on HaCaT cell proliferation and the regulatory protein expression were evaluated and the molecular mechanisms of action of metformin in the treatment of psoriasis were investigated.

## Materials and methods

### Cells and reagents

The HaCaT cell line was purchased from the American Type Culture Collection (Manassas, VA, USA). Metformin hydrochloride was purchased from Sigma-Aldrich (St. Louis, MO, USA). The cell proliferation and cytotoxicity assay kit (Cell Counting Kit-8; CCK-8) was purchased from Dojindo (Kunamoto, Japan). p-AMPK α1 and p-MAPK 1/2 antibodies were purchased from Abcam (Cambridge, MA, USA). The quantitative automatic microplate reader (model no., 2010) was purchased from Anthos Labtec Co., Ltd. (Salzburg Austria). The study was approved by the ethics committee of Shandong University (Jinan, China).

### Metformin treatment

HaCaT cells were cultured in Dulbecco’s modified Eagle’s medium (DMEM) with 10% fetal bovine serum, 100 U/ml penicillin and 100 μg/ml streptomycin at 37°C in a 5% CO_2_ humidified and sterile environment. HaCaT cells during the logarithmic growth phase were collected and inoculated in 96-well (1×10^4^ cells/well) or 6-well plates (3×10^5^ cells/well). Two groups, specifically, control (without metformin) and metformin (50 mM metformin) were established. After 24 h of inoculation, the culture medium in the metformin group was replaced with DMEM containing metformin to maintain the metformin concentration at 50 mM. An equal volume of phosphate-buffered saline (PBS) was added to the control group.

### Cell morphology observation

Following 24 h of 50 mM metformin treatment, the morphology of HaCaT cells was observed under an inverted microscope (Olympus BX-51; Olympus optical Co., Ltd., Tokyo, Japan).

### CCK-8 assay

Following 3–5 stable passages, HaCaT cells in the logarithmic phase were inoculated in 96-well plates. Cell culture medium (100 μl DMEM; Invitrogen, Carlsbad, CA, USA) and 100 μl metformin were added to the center of 60 wells. Following cross mixing, cells were cultured at 37°C in 5% CO_2_ for 24, 48 and 72 h. CCK-8 solution was added and the optical density (OD) values were detected at 450 nm using a quantitative automatic microplate reader (model no. 2010; Anthos Labtec Co., Ltd.). Cell survival rates at the various treatment times were calculated and the cell survival curve was drawn. The cell survival rate (%) was calculated using the following formula: (OD_metformin_ - OD_control_)/(O_Dcontrol_ - OD_metformin_) × 100.

### Western blot analyses

Total protein was extracted from each sample and antibody incubation was performed according to the instructions of the manufacturer of the one-step rapid WB kit (rabbit; Shanghai Biological Engineering Co., Ltd., Shanghai, China) using antibodies against p-AMPK (1:200) and p-extracellular signal-regulated kinase (ERK1/2; 1:250). The ultra-sensitive enhanced chemiluminescence kit (Biyuntian Biotechnology Institute, Beijing, China) was used for color development. The membrane was incubated with the ECL Plus A and Plus B reagents for 2 min at room temperature. The membrane was developed in the dark. The developed films were scanned using the AlphaImager gel imaging systems (AlphaImager, Santa Clara, CA, USA). The western blot images were then analyzed using Quantity One software (Bio-Rad Laboratories, Hercules, CA, USA). β-actin was used as an internal control. The relative absorbance ratios of p-AMPK to β-actin and p-ERK1/2 to β-actin were defined as the relative values of p-AMPK and p-ERK1/2, respectively.

### Statistical analyses

All experimental data are presented as the mean ± standard deviation. SPSS statistical software (v13.0; SPSS, Inc., Chicago, IL, USA) was used for analysis. One-way analysis of variance was used for mean comparisons. P<0.05 was considered to indicate a statistically significant difference.

## Results

### Effect of metformin on HaCaT cells

**Figure 1:**
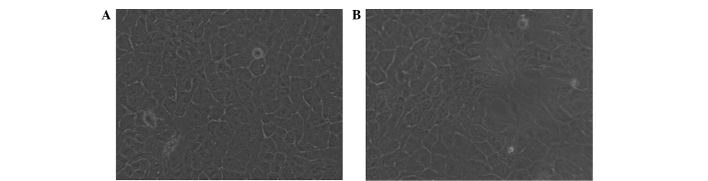
Morphology analysis of HaCaT cells following metformin treatment. Cells were observed under an inverted microscope (magnification, ×200). (A) Untreated HaCaT cells and (B) HaCaT cells treated with 50 mM metformin for 24 h. HaCat, human immortalized keratinocytes.

To determine the effects of metformin treatment on the morphology of HaCaT cells, cells were observed under an inverted microscope after 24 h of treatment. The untreated HaCaT cells were in adherent growth and arranged in cobblestone and mosaic shapes. Cells were flat and polygonal with a clear boundary, abundant cytoplasm, and a round or oval nucleus (Fig. 1A). The cells in the metformin group were treated with 50 mM metformin for 24 h. The sizes of treated cells were slightly smaller compared with those of the untreated cells (Fig. 1B). The sizes of cell granules and vacuoles were increased and a number of cells had been killed by the treatment (Fig. 1B).

**Figure 2:**
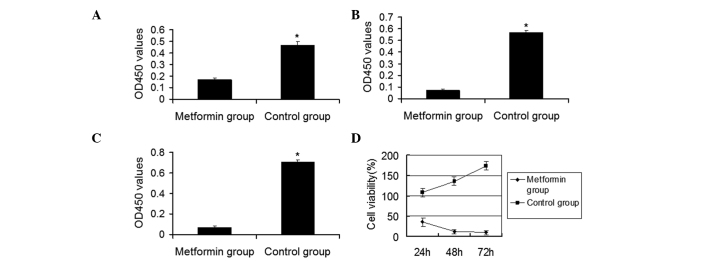
Cell proliferation analysis of HaCaT cells following metformin treatment. After treatment with 50 mM metformin for 24, 48 and 72 h, cell viability was detected by a CCK-8 kit. The OD values at 450 nm of each group were measured and the cell viability was calculated. OD value at 450 nm of each group at (A) 24, (B) 48 and (C) 72 h. (D) Cell viability of each group at 24, 48 and 72 h following metformin treatment. Cell survival rate (%) was calculated using the following formula: (OD_metformin_ - OD_control_)/(OD_control_ - OD_metformin_) × 100. Experiments were conducted three times and data are expressed as the mean ± standard deviation. ^*^P<0.05, vs. metformin group. HaCat, human immortalized keratinocytes; OD, optical density; CCK-8, cell proliferation and cytotoxicity assay kit-8.

To investigate cell proliferation following metformin treatment, the CCK-8 cell viability assay was performed. As shown in Fig. 2, the survival fractions of HaCaT cells treated with metformin were 36.18, 12.70 and 10.12% at 24, 48 and 72 h, respectively, and were significantly lower compared with those of the untreated control (P<0.05). With extended metformin treatment time, HaCaT cell survival rates gradually decreased.

Collectively, these results suggest that metformin induces changes in HaCaT cell morphology and inhibits HaCaT cell proliferation *in vitro*.

### Effect of metformin on AMPK and ERK1/2 protein expression in HaCaT cells

**Figure 3:**
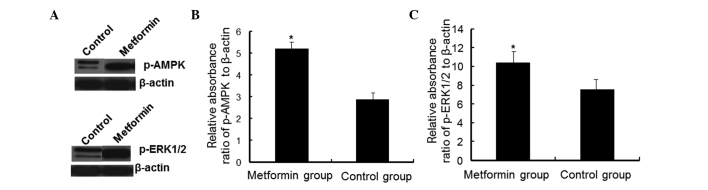
Analysis of p-AMPK and p-ERK1/2 expression in HaCaT cells following metformin treatment. Expression of p-AMPK and p-ERK1/2 in HaCaT cells was evaluated by western blot analysis 24 h after metformin treatment. β-actin was used as the internal control. (A) Western blot analysis results of p-AMPK and p-ERK1/2 expression levels and the relative absorbance ratio of (B) p-AMPK to β-actin of each group and (C) p-ERK1/2 to β-actin of each group. Data are presented the mean ± standard deviation of three independent experiments. ^*^P<0.05, vs. the control group. p, phosphorylated; HaCat, human immortalized keratinocytes; AMPK, adenosine monophosphate-activated protein kinase; ERK1/2, extracellular signal-related kinase 1/2.

To determine whether the MAPK signaling pathway was activated by metformin, the levels of p-AMPK and p-ERK1/2 were detected by western blot analysis. As shown in Fig. 3A and B, in the cells treated with PBS, the mean absorbance ratio of p-AMPK relative to β-actin in HaCaT cells was 2.856±0.323. However the mean absorbance ratio of the cells treated with metformin was 5.198±0.625. The expression levels of p-AMPK between these two groups were identified to be significantly different (P<0.05; Fig. 3).

As shown in Fig. 3A and C, the mean absorbance ratio of p-ERK1/2 relative to β-actin in the untreated HaCaT cells was 7.550±1.087, but the ratio in the cells treated with metformin was 10.430±1.217, with a significant difference between the two groups (P<0.05). These results indicated that the above proteins were activated following metformin treatment for 24 h. After 24 h of metformin treatment, the expression levels of p-AMPK and p-ERK1/2 markedly increased. This suggests that AMPK and ERK1/2 proteins were phosphorylated due to metformin treatment. Therefore, metformin inhibited HaCaT cell proliferation, possibly via a mechanism associated with the activation of the MAPK signaling pathway.

## Discussion

In the present study, HaCaT cells were used as an *in vitro* model of psoriatic keratinocyte proliferation to analyze the effects of metformin on HaCaT cell proliferation and investigate the possible mechanisms of its treatment for psoriasis. The CCK-8 assay showed that metformin significantly inhibited the proliferation of HaCaT cells *in vitro*. Western blot analysis results suggested that metformin stimulated AMPK and ERK1/2 phosphorylation in HaCaT cells. These observations indicate that metformin activates AMPK in the ERK1/2 signaling pathway and regulates its downstream gene expression to inhibit cell proliferation. In addition, following metformin treatment, the levels of p-AMPK protein were significantly increased.

ERK1/2 is an important signaling pathway in the MAPK family that regulates cell growth and differentiation. The ERK1/2 signaling pathway is closely associated with psoriasis, but whether there is phosphorylation of ERK1/2 in psoriasis lesions remains controversial. ERK1/2 activation is dependent upon stimulus intensity, thus the intensity of ERK activation is likely to affect the response (18). It has been previously reported that the ERK1/2 signaling pathway exhibits dual effects in promoting HaCaT cell proliferation through epidermal growth factors whose effects on HaCaT cell proliferation and activation are associated with ERK signal intensity (19). However, overactivation of the ERK signal inhibits cell proliferation. For example, Pumiglia *et al*(20) reported that activation of ERK inhibits CDK activity and induces cell cycle arrest through induction of the CDK inhibitor p21^Cip1/WAF1^. Wang et al (21) also observed that persistent activation of ERK lead to cell cycle arrest. Tang *et al*(22) showed that ERK activation partially contributed to p21Cip1/WAF1 induction. In the present study, we found that after metformin treatment, HaCaT cell proliferation was inhibited while p-ERK was upregulated. Therefore we suggest that the inhibition of metformin on HaCaT cells is mediated by ERK activation.

In conclusion, the MAPK signal transduction pathway is important in mammalian cells. In the present study, metformin enhanced the expression of p-ERK1/2, suggesting that the mechanism by which metformin inhibits cell proliferation may be associated with activation of the MAPK signaling pathway. However, the mechanisms associated with the transduction of stimulus signals via the ERK1/2 signaling pathway and whether other signaling pathways are involved, requires further study.
